# Growth patterns and life-history strategies in Placodontia (Diapsida: Sauropterygia)

**DOI:** 10.1098/rsos.140440

**Published:** 2015-07-08

**Authors:** Nicole Klein, James M. Neenan, Torsten M. Scheyer, Eva Maria Griebeler

**Affiliations:** 1 State Museum of Natural History Stuttgart, Rosenstein 1, 70191 Stuttgart, Germany; 2 Palaeontological Institute and Museum, University of Zurich, Karl Schmid-Strasse 4, 8006 Zürich, Switzerland; 3 Department of Ecology, Zoological Institute, University of Mainz, 55099 Mainz, Germany

**Keywords:** growth record, logistic growth model, von Bertalanffy growth model, growth marks, non-annual rest lines

## Abstract

Placodontia is a clade of durophagous, near shore marine reptiles from Triassic sediments of modern-day Europe, Middle East and China. Although much is known about their primary anatomy and palaeoecology, relatively little has been published regarding their life history, i.e. ageing, maturation and growth. Here, growth records derived from long bone histological data of placodont individuals are described and modelled to assess placodont growth and life-history strategies. Growth modelling methods are used to confirm traits documented in the growth record (age at onset of sexual maturity, age when asymptotic length was achieved, age at death, maximum longevity) and also to estimate undocumented traits. Based on these growth models, generalized estimates of these traits are established for each taxon. Overall differences in bone tissue types and resulting growth curves indicate different growth patterns and life-history strategies between different taxa of Placodontia. *Psephoderma* and *Paraplacodus* grew with lamellar-zonal bone tissue type and show growth patterns as seen in modern reptiles. Placodontia indet. aff. *Cyamodus* and some Placodontia indet. show a unique combination of fibrolamellar bone tissue regularly stratified by growth marks, a pattern absent in modern sauropsids. The bone tissue type of Placodontia indet. aff. *Cyamodus* and Placodontia indet. indicates a significantly increased basal metabolic rate when compared with modern reptiles. Double lines of arrested growth, non-annual rest lines in annuli, and subcycles that stratify zones suggest high dependence of placodont growth on endogenous and exogenous factors. Histological and modelled differences within taxa point to high individual developmental plasticity but sexual dimorphism in growth patterns and the presence of different taxa in the sample cannot be ruled out.

## 1. Background

### 1.1 Placodontia

**Figure 1.**
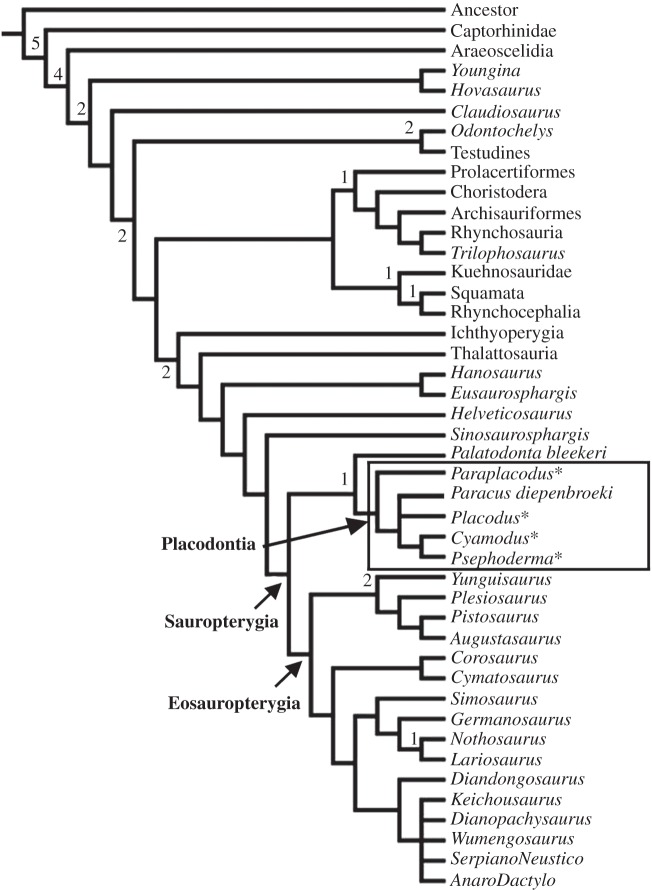
Phylogenetic relationships within Diapsida, including Sauropterygia and Placodontia. The asterisks mark the histologically studied members of Placodontia. Cladogram modified from Neenan *et al.* [13] and Klein & Scheyer [108]. Bremer support values are given above the nodes.

Placodontia are a basal group of Sauropterygia, a diverse radiation of diapsid marine reptiles that ranged from the late Lower Triassic until the end of the Cretaceous [1–3]. The Triassic radiation of Sauropterygia consisting of the shallow marine Placodontia, Pachypleurosauria, and Nothosauria as well as the more open marine Pistosauria; conversely, the Jurassic and Cretaceous seas were ruled by the open marine Plesiosauria (figure 1). The latter had a global distribution, whereas most Triassic representatives were largely restricted to the epicontinental seas of the Tethys. Both studies of morphology (summarized in [1]) and bone histology [4–11] of Triassic Sauropterygia document a high variety of ecologies, life histories and feeding strategies, which enabled them to live contemporaneously in the same habitats.

Placodontia ranged from the lower Middle Triassic (Anisian) until the Late Triassic (Rhaetian). They can be divided into a non-armoured placodontoid grade, which was recently resolved as a monophyletic group [12], and a heavily armoured monophyletic Cyamodontoidea [1,13]. Placodonts shared some unique morphological features, among which are a massive, akinetic skull and a reduced but specialized dentition enabling a durophagous diet (summarized in [14,15]). Microanatomical data support an aquatic lifestyle for all Placodontia with varying but slow, restricted swimming skills as bottom dwellers [11]. Placodontia lived in shallow marine environments in the Germanic Basin and along the coasts of the epicontinental seas of the western and eastern Tethys (modern-day Europe/Middle East and South China) [1,16,17].

### 1.2 Growth records in fossil vertebrates

The study of the growth record of fossil vertebrates is the only direct possibility to gather data on major life-history traits of extinct animals. Skeletochronology, a field of bone histology that aims at the study and practical use of cyclical growth marks, provides information on: age and size at sexual maturity, age at which maximum individual size was achieved, the length of an individual's life/age at death and maximum growth rates. Studying the growth record of an individual or of an ontogenetic growth series reveals series of ages at respective body masses/bone lengths from which statistically assured growth curves can be established.

The construction of these growth curves not only allows the estimation of growth traits that are documented in the histological record but also of those that are undocumented. It thus reveals important insights into life history at the individual and population level, the latter by averaging traits across several individuals [18–21]. Finally, given sufficient data, a growth pattern and strategy for a taxon can be deduced. In modern amphibians and reptiles, the onset of sexual maturity is reached years before maximum size is attained [18,22]. In most living birds and in small mammals (not true for some birds and for larger mammals such as the elephant bull), however, the onset of sexual maturity and attainment of maximum size occurs largely simultaneously [18,23].

Comparison of such life-history data between taxa can lead to the identification of underlying heterochronic processes, which are mainly changes in growth (changes in growth rate or in the onset or offset of character timing) and contribute to the general understanding of the evolution of life histories [24].

Patterns in ageing, maturation and growth have been studied using bone histology in many extinct tetrapod groups, such as temnospondyls [21,25], archosaurs [26], dinosaurs [19,27–31] and early synapsids [32,33]. Many of these studies have detected combinations of bone tissues and growth patterns, implying life-history strategies not in existence today as well as developmental pathways not known in any living taxon.

It is the aim of this paper to describe, compare and interpret in detail the growth patterns and strategies documented in five placodont taxa (23 individuals), and to establish information on their ageing and maturation. Thus, the growth record of specimens will be histologically evaluated and growth models established. Growth models are used to validate life-history traits documented in the histological record and to estimate undocumented traits. Finally, based on growth models, generalized estimates of these traits are established for each taxon.

### 1.3 Growth marks and modelling

The annual periodicity of growth cycles was tested and confirmed in many living vertebrates [34], such as amphibians [35], iguanines [36,37], lacertids [38,39], varanids [40–42], turtles [43–47], crocodylians [48–50] and mammals [51–53]. The deposition of growth marks follows external and internal rhythms, which are often synchronized. However, seasonality and other environmental factors seem to remain the main trigger for the deposition of cyclical growth marks [54].

Fitting different growth models to a series of ages and respective body masses/bone lengths derived from an annual growth record preserved in a single bone is an objective method to find the statistically best growth curve for the sample. From the growth curves, life-history traits of the individual can be estimated, as was exemplified by Griebeler *et al.* [27] for sauropodomorph dinosaurs and by Klein & Griebeler [55] for the eosauropterygian *Simosaurus*. Standard growth models basically differ in the position of the inflection point, which is assumed to coincide with sexual maturity in amphibians and reptiles (summarized in [56]).

However, growth models are not taxon-specific and individuals of a taxon can follow different curves owing to individual differences in growth rate (e.g. genetic constitution), and environmental factors (e.g. ambient temperature, water, food availability) [57].

### 1.4 Institutional abbreviations

IGWH: Institute of Geosciences of the Martin-Luther-University Halle-Wittenberg, Germany
MB.R: Museum of Natural History, Leibniz Institute for Research on Evolution and Biodiversity at the Humboldt University Berlin, Germany
MHI: Muschelkalkmuseum Ingelfingen, Germany
PIMUZ: Palaeontological Institute and Museum of the University of Zurich, Switzerland
SMNS: Stuttgart State Museum of Natural History, Germany

## 2. Material

A complete list of the studied material is presented in table 1 and a detailed description of the morphology, histology and microanatomy of the bones is given in Klein *et al*. [11]. All studied bones were found isolated without any diagnostic material attached or associated. Bones from localities of the Alpine Triassic realm (table 1) had been compared to diagnostic material and could be assigned to *Psephoderma* [58–60] and *Paraplacodus* [1,61,62]. The assignment of isolated bones from Muschelkalk localities (Germanic Basin; table 1) is problematic owing to the lack of diagnostic material and incomplete preservation. For example, neither a *Cyamodus* nor a *Placodus* skull has yet been found together with a humerus in a Muschelkalk locality [1,63,64]. Taxonomical assignment of studied bones to placodonts is based on morphological [1,62,65,66] and histological comparison [4–8,10,67,68]. Studied bones are thus only tentatively assigned to a taxon, and bones combined here in a taxon might in fact include several taxa [11]. However, the assignment of studied bones to Placodontia is unequivocal [11]. *Horaffia kugleri* is so far only known from humeri, which show a distinct pachyosteosclerosis and a comparable histology to that of Placodontia indet. aff. *Cyamodus* [11,69], so it is thus interpreted as a marine reptile with placodont affinities.

**Table 1.** List of material and summary of bone histological data. (Bone length and perimeter (in mm), localities, stratigraphical information, bone tissue type, growth mark count (visible and reconstructed), age at the onset of sexual maturity and bone tissue in the outer cortex of sampled placodont bones and *Horaffia* *kugleri*. The number in parenthesis gives the years at which the individual died. This is the case when the outer cortex shows an incomplete growth cycle. bl, bone length; btt, bone tissue type; EFS, external fundamental system; FLB, fibrolamellar bone; LZB, lamellar-zonal bone; litho, lithostratigraphical group; sm, sexual maturity; p, perimeter; plex., plexiform; r, radial; recon. gm, number of reconstructed growth marks of the inner cortex; strati., stratigraphic age; LAGs, lines of arrested growth.)

taxon spec. no.	bl	p	strati./litho.	locality	btt	visible annual gm	recon. gm	age at onset sm.	outer cortex	ontogenetic stage
*Psephoderma*										
PIMUZ A/III 1476 humerus	55.4	20	Rhaetian	Tinzenhorn, Fil da Stidier, Filisur (Grissons, Switzerland)	LZB	25	3	13	EFS	adult, fully grown
PIMUZ A/III 0735 femur	85.6	30	Rhaetian	Chrachenhorn, Monstein (Grissons, Switzerland)	LZB	10	2	?5 o. ?7	EFS	adult, fully grown
Placodontia indet. aff. *Cyamodus*										
SMNS 59831 humerus	>120	110	Ladinian (Upper Muschelkalk)	Weiblingen (B-Württemberg, Germany)	r. to plex. FLB	7(8)	0	?	?annulus or subcycle	adult, ?close to fully grown
SMNS 15937 humerus	222	105	Ladinian (Upper Muschelkalk)	Crailsheim/Tiefenbach (B-Württemberg, Germany)	r. to plex. FLB	7(8)	0	?	zone	adult, ?close to fully grown
MHI 2112–6 humerus	∼205	96	Ladinian (Muschelkalk/ Keuper Grenzbonebed)	Satteldorf-Barenhalden (B-Württemberg, Germany)	r. to plex. FLB	3(4)	0	?	annulus	adult
SMNS 54569 humerus	>129	92	Ladinian (Upper Muschelkalk)	Hegnabrunn (Bavaria, Germany)	r. to plex. FLB	5 (6)	0	?	?annulus or subcycle	adult
SMNS 54582 humerus	>85	79	Ladinian (Muschelkalk/ Keuper)	Obernbreit (Bavaria, Germany)	r. to plex. FLB	4 (5)	0	?	zone	adult
SMNS 15891 humerus	175	76	Ladinian (Upper Muschelkalk)	Crailsheim/Tiefenbach (B-Württemberg, Germany)	r. to plex. FLB	5 (6)	0	?	zone	adult
MHI 697 humerus	>111	82	Ladinian (Muschelkalk/ Keuper Grenzbonebed)	Satteldorf-Barenhalden (B-Württemberg, Germany)	r. to plex. FLB	3 (4)	0	?	zone	adult
MHI 1096 humerus	>67	54	Ladinian (Upper Muschel-kalk)	Ummenhofen (B-Württemberg, Germany)	r. to plex. FLB	?5 (?6)	0	?	zone	juvenile
*Paraplacodus*										
PIMUZ T5845 humerus	104.5	∼39	Anisian/Ladinian (Besano Formation, Middle Grenzbitumen-zone)	Monte San Giorgio, Meride (Ticino, Switzerland)	LZB	7 (8)	1	?	regularly spaced LAGs	adult
PIMUZ T5845 femur	89.1	∼25	Anisian/Ladinian (Besano Formation, Middle Grenzbitumen-zone)	Monte San Giorgio, Meride (Ticino, Switzerland)	LZB	9	1	?	regularly spaced LAGs	adult
Placodontia indet. group I										
MB.R. 454 humerus	120	53	Anisian (Lower Muschelkalk)	Ohrdruf (Górny Ślask, Poland)	FBL	5 (6)	2	?	annulus	adult
IGWH 9 humerus	68	36.5	Anisian (Lower to Middle Muschelkalk)	Freyburg, River Unstrut valley (East-Germany)	FBL	6 (7)	0	3	annulus	adult
SMNS 84545 femur	>160	105	Ladinian (Muschelkalk/Keuper)	Hegnabrunn (Bavaria, Germany)	FBL	23 (EFS 8)	1–2	?	EFS	adult, fully grown
IGWH 23 femur	>57	55.3	Anisian (Lower to Middle Muschelkalk)	Freyburg, River Unstrut valley (East-Germany)	FBL	9 (10)	∼9	5–613	zone	?adult
Placodontia indet. group II										
MB.R. 814.2 femur	>100	∼127	Anisian (Lower Muschelkalk)	Górny Ślask, Poland	FBL	13 (14)	0	3	annulus	adult
MB.R. 961 femur	>145	89	Ladinian (Upper Muschelkalk)	Bayreuth (Bavaria, Germany)	FBL	8 (9) (EFS 2)	0	?	EFS	adult, fully grown
MB.R. 812 femur	>120	70.6	Anisian (Lower Muschelkalk)	Opatowitz (Górny Ślask, Poland)	FBL	5 (6)	0	4	zone	adult
*Horaffia kugleri*										
MHI 2112-1 humerus	77.6	50	Ladinian (Muschelkalk/Keuper Grenzbonebed)	Satteldorf-Barenhalden (B-Württemberg, Germany)	plex. FBL	2 (3)	0	?	zone	juvenile
SMNS 84816 humerus	8.35	43	Ladinian (Muschelkalk/Keuper Grenzbonebed)	Crailsheim (B-Württemberg, Germany)	plex. FBL	3 (4)	0	?	zone	juvenile
MHI 2112-2 humerus	>111	65	Ladinian (Muschelkalk/Keuper Grenzbonebed)	Vellberg-Eschenau (B-Württemberg, Germany)	plex. FBL	2 (3)	0	?	zone	juvenile
MHI 2112-4 humerus	152	82	Ladinian (Muschelkalk/Keuper Grenzbonebed)	Obersontheim-Ummenhofen (B-Württemberg, Germany)	plex. FBL	4 (5)	0	?3	zone	adult

### 2.1 Methods

**Figure 2.**
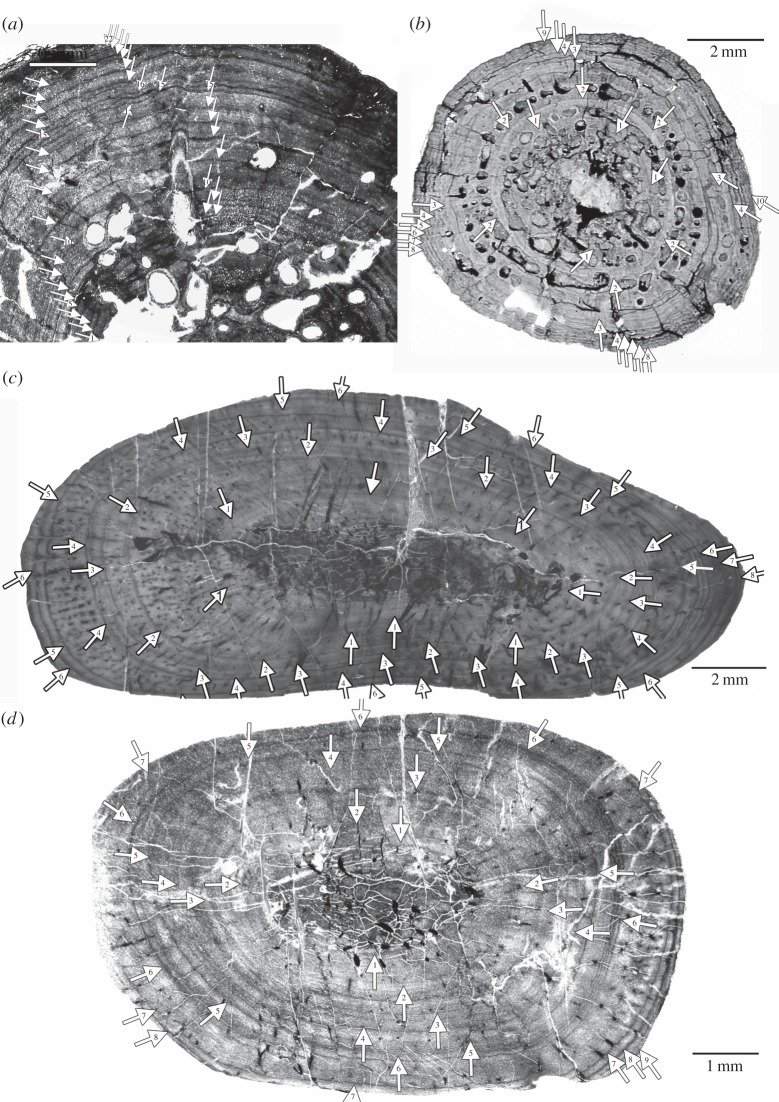
(*a*) Growth record traced in part of the scanned cross section of *Psephoderma* humerus (PIMUZ A/III 1476) and (*b*) scanned cross section of *Psephoderma* femur (PIMUZ A/III). (*c*) Growth record traced in the scanned cross section of *Paraplacodus* humerus (PIMUZ T5845) and (*d*) scanned cross section of *Paraplacodus* femur (PIMUZ T5845).

**Figure 3.**
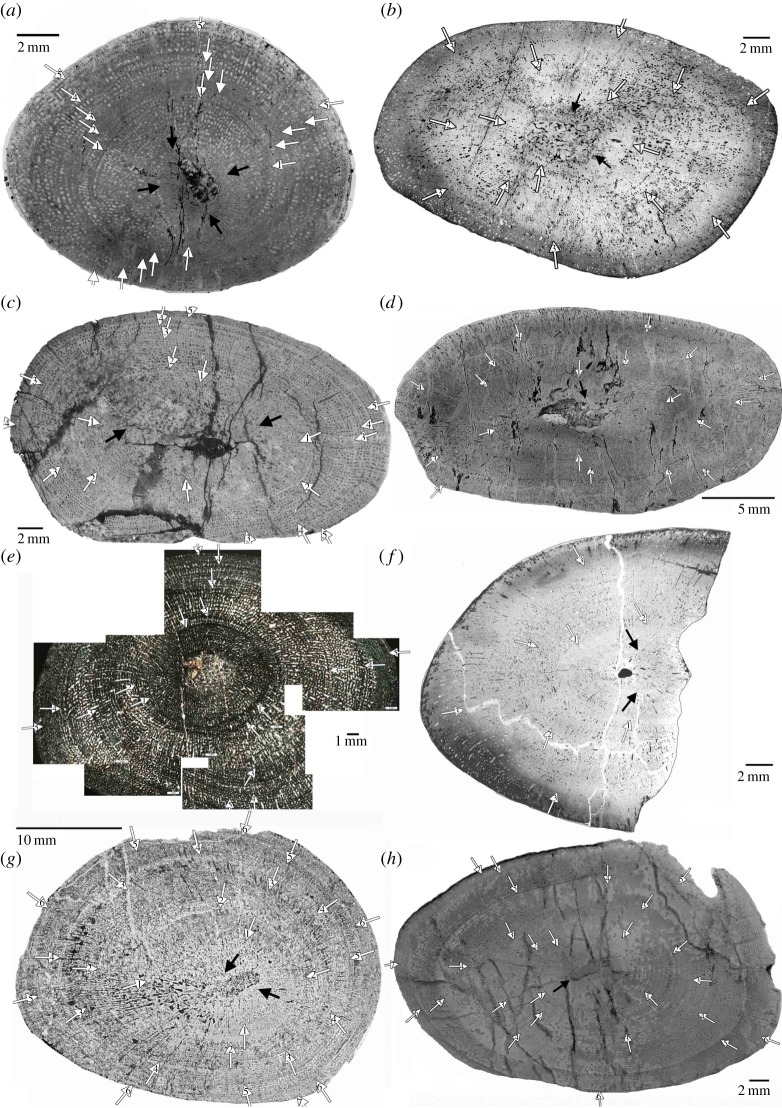
Growth record traced in scanned cross sections of humeri of Placodontia indet. aff. *Cyamodus*. (*a*) MHI 1096, (*b*) MHI 697, (*c*) SMNS 15891, (*d*) SMNS 54582, (*e*) composite of part of the cross section in polarized light of humerus SMNS 54569, (*f*) MHI 2112–6, (*g*) SMNS 15937 and (*h*) SMNS 59831.

**Figure 4.**
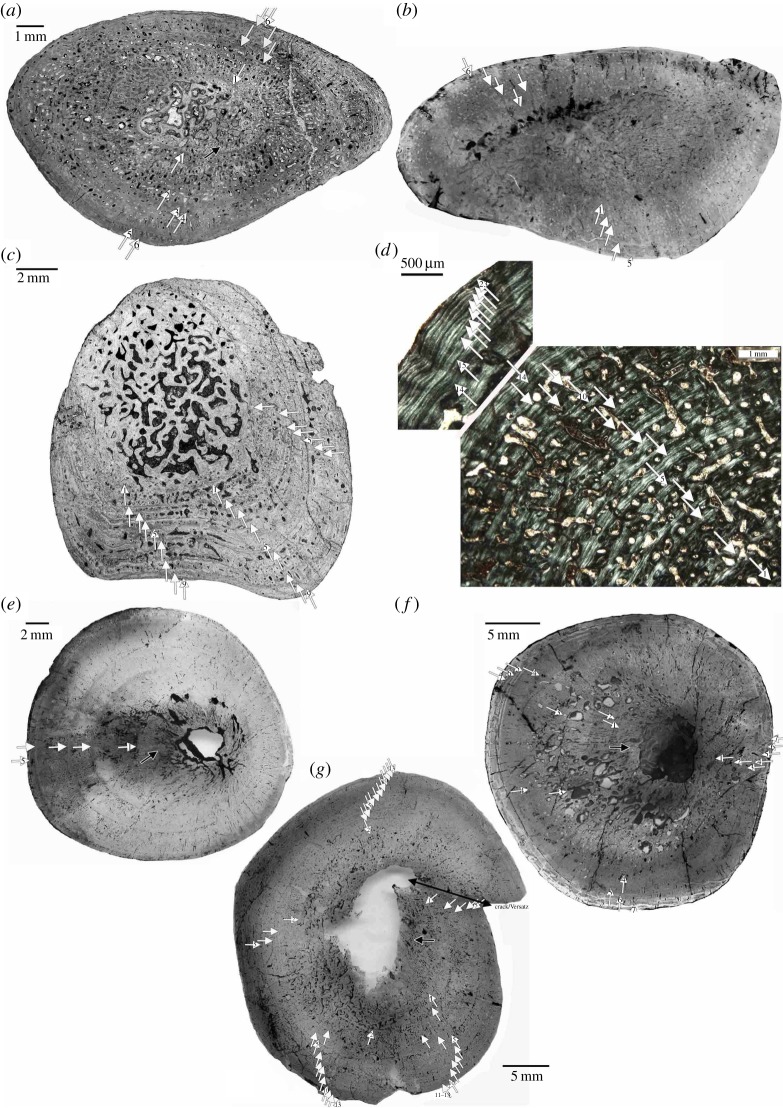
*a*–*d*, Growth record traced in scanned cross sections of bones of Placodontia indet. group I. (*a*) Humerus IGWH 9, (*b*) humerus MB.R. 454, (*c*) femur IGWH 23, (*d*) femur SMNS 84545 and (*e*–*g*) growth record traced in scanned cross sections of femora of Placodontia indet. group II. (*e*) MB.R. 812, (*f*) MB.R. 961, (*g*) MB.R. 814.2.

**Figure 5.**
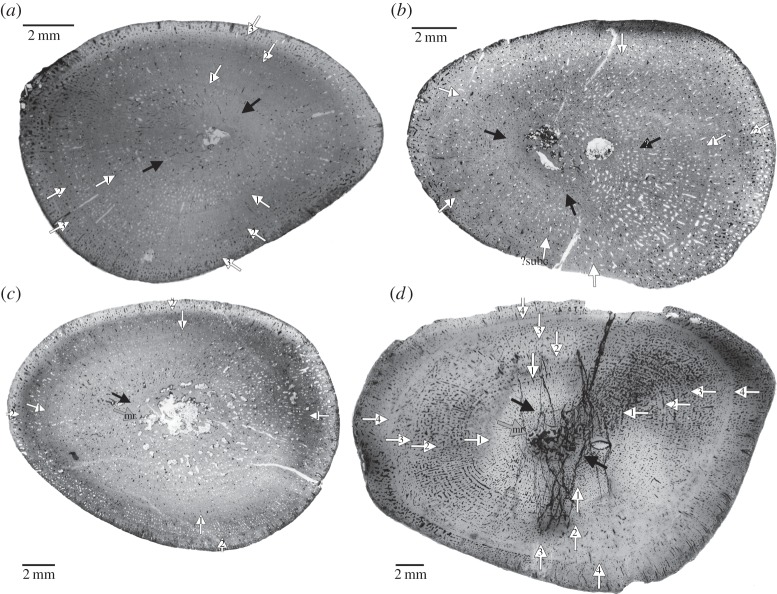
Growth record traced in scanned cross sections of humeri of *Horaffia kugleri*. (*a*) SMNS 84816, (*b*) MHI 2112–1, (*c*) MHI 2112–2, (*d*) MHI 2112–4.

The midshaft area of placodont long bones contains the growth centre and has the most complete growth record preserved. The thin sections were produced by cutting an entire cross section from the midshaft region and their production followed standard petrographic methods as outlined, for example, in Klein & Sander [30]. Thin sections were studied and photographed with a Leica® DMLP compound polarizing microscope, equipped with a digital camera (Leica DFC 420C) and a Leica DM 750P compound polarizing microscope equipped with a digital Leica ICC50HD camera. Cross sections were scanned with an Epson V740 PRO high-resolution scanner. The bone histological terminology follows Francillon-Vieillot *et al.* [70]. Annual growth cycles, which are the basis for growth modelling, were marked in the scanned cross sections or on microscopic images with Adobe Photoshop CS5.1 (figures 2–5). These images were used to measure the distance of annual growth marks from the centre of each medulla. Then the corresponding bone length was calculated for each growth mark (for details, see [27,55]). Only growth marks that can be followed across the entire section are interpreted as annual growth marks herein.

**Figure 6.**
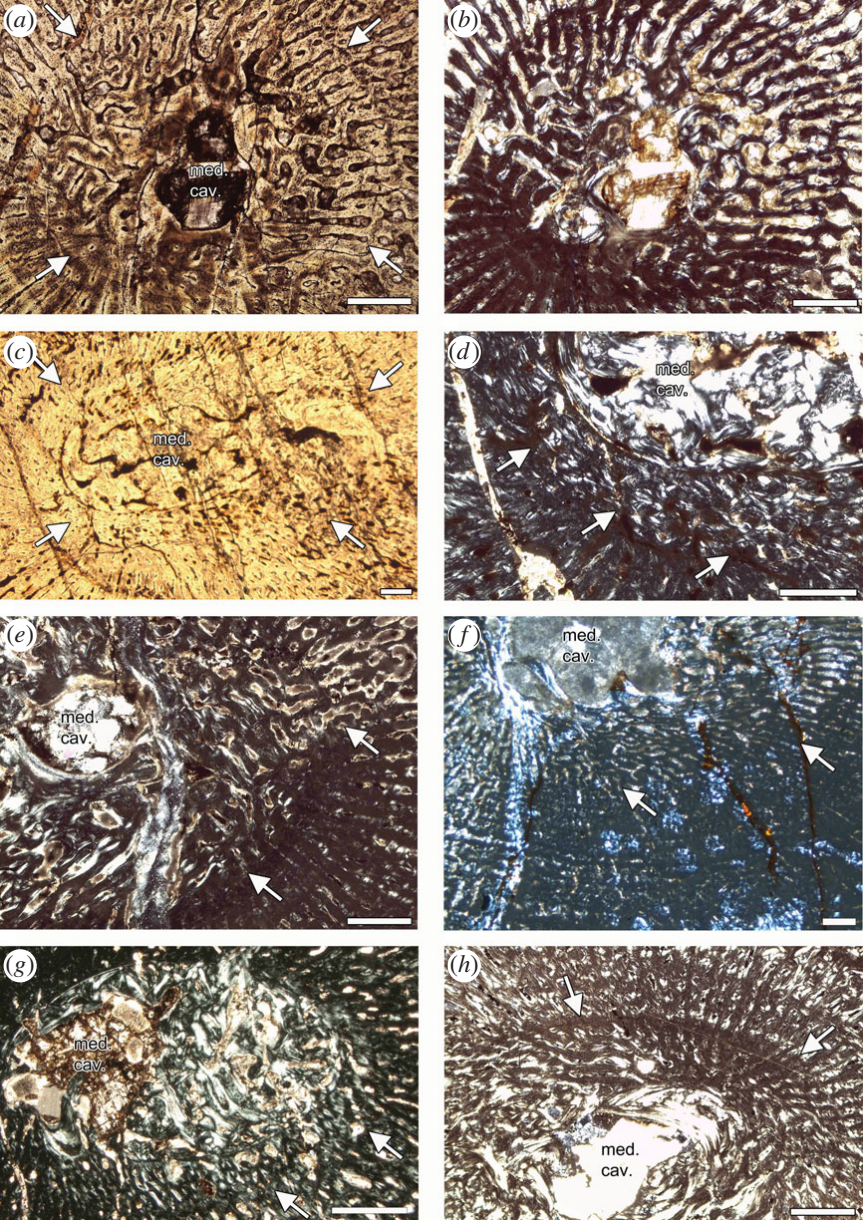
Perinatal bone tissue in Placodontia indet. aff. *Cyamodus* humeri. Images (*a*) and (*c*) viewed in normal transmitted and (*b*,*d*–*h*) in cross-polarized light. (*a*,*b*) Centre of cross section in MHI 1096, (*c*) centre of cross section in MHI 697, (*e*) centre of cross section in MHI 2112–6, (*f*) centre of cross section in SMNS 59831, (*g*) centre of cross section in SMNS 54569, and (*h*) centre of cross section in a *Horaffia kugleri* humerus (SMNS 84816). The arrows always mark the end of the perinatal bone tissue. Scale bar is 0.5 mm.

In *Psephoderma*, *Paraplacodus* and some Placodontia indet., an unknown number of inner growth marks are lost owing to the expansion of the medulla and their numbers were extrapolated (table 1) [27,30,55]. A reconstruction of inner annual growth cycles was not necessary when perinatal bone tissue was present (see description below; figure 6). The area containing perinatal bone tissue is not counted as a separate annual growth cycle but as part of the first annual growth cycle.

### 2.2 Modelling growth

To construct a mass-based growth curve for a single individual, the relationship between local bone apposition rate and body mass gain is taken into account [27,55]. Mass-based growth curves are more informative because the position of the inflection point on the age axis (and thus onset of sexual maturity) is predicted by standard growth models from the mass axis [56]. However, reliable mass reconstruction is not possible for Placondontia owing to poor preservation, incompleteness of specimens and the presence of armour and pachyostosis. Thus, in this study only bone length can be used to construct growth curves. However, the principle for constructing length-based growth curves is the same as for mass-based growth curves. Minimal shaft circumference increases proportionally with bone length and the local bone apposition rate is closely tied to gain in body mass or size. Thus, each growth mark laid down during ontogeny is linked to a respective bone length and in turn to a specific body mass. The series of growth mark number versus corresponding bone length is used here to establish mathematical growth models for each specimen using the method introduced by Griebeler *et al*. [27] and Klein & Griebeler [55].

For all Placodontia indet. aff. *Cyamodus* and Placondontia indet., growth curves were established from the ventral and postaxial sides of the bone, as the distance between two respective growth cycles considerably varies between these loci. However, as postaxial and ventral growth curves were consistent with respect to the type of the best model, the presentation of results and the discussion is limited to the postaxial growth curves. For all other bones, the growth record was used from the bone side where it was best preserved.

Four sigmoidal growth models were considered: von Bertalanffy (vBGM), Gompertz (GGM), logistic (LGM) and Chapman-Richards (CRGM). In addition, a linear model (*L*(*t*)=*L*_0_+*g*⋅*t*) was also fitted to each of the histological growth series before testing the four sigmoidal models. This was done to test whether growth series only cover the quasi-linear phase of growth (around the inflection point). If so, no information on sigmoidal/asymptotic growth is preserved [71]. All models relate the bone length of an individual (*L*(*t*)) to its age (*t*>0). In all models, *L*_0_ is a (non-zero) initial bone length/bone length of the perinate, and *g* is the growth rate. In all sigmoidal models, *A* is the asymptotic bone length of the individual. As the vBGM shows no inflection point when bone length is plotted against age, the age at which 30% of bone length was reached was used to estimate the age at the onset of sexual maturity. The application of the GGMs and CRGMs to placodonts revealed either unreliable growth models (e.g. a negative *L*_0_) or the growth curve fitting procedure failed. Thus, both models are omitted in the remaining part of the study.

In the case that more than one growth model was successfully fitted to an ontogenetic growth series, the statistically best growth model was selected based on the *absolute* (residuals) and *relative* goodness of fit of models (Akaike information criterion, AIC) [55,72,73] (table 2). All regression analyses were carried out with the statistical software, R (v. 3.0.2, http://www.R-project.org). The function ‘nls’ from the nls package (nonlinear regression analysis) was applied to fit growth models to ontogenetic growth series and to calculate AIC values of models.

**Table 2.** Results of growth curve fitting for Placodontia. (Only the statistically best growth models are shown. From the fitted growth curves, we estimated the age at death of the individual (AD), and only for sigmoidal models the age at which it (would have or had) achieved asymptotic size (AA) and the age it reached sexual maturity (ASM). Growth curve fitting followed the procedure described in [27,55]. Overall, three growth models were successfully fitted to growth series of placodonts: linear growth model (LM, tests whether the growth record only covers the linear phase of growth, *L*(*t*)=*L*_0_+*g*×*t*), von Bertalanffy growth model (vBGM, *L*(*t*)=*A*−(*A*−*L*_0_) exp(−*g*×*t*)), and logistic growth model (LGM, *L*(*t*)=*L*_0_+*A*/(1+exp (−*g*×(*t*−*t*_*i*_)))), where *L*(*t*) is femur length at age *t*; *L*_0_, femur length of the perinate (*t*=0); *g*, growth rate; *A*, asymptotic femur length (only vBGM and LGM); and *t*_*i*_, age at which the inflection point is observed (only LGM). Goodness of fit of growth series to models was assessed by the Residual standard error (Res. s.e., degrees of freedom (d.f.)) and the AIC value (Akaike information criterion, [76], d.f.). In order to find the best statistical growth model for a specimen, we identified its model with the lowest AIC value, assessed relative goodness of fit of different candidate models by calculating ΔAIC values (AIC of the candidate model—AIC of the model with the lowest AIC) and selected the best statistical model following the approach in Burnham & Anderson [73]. To document whether the sigmoidal model is supported over the linear model, we show Δ Res_LM_ (i.e. difference in Res. s.e. between the sigmoidal model and the linear model) and ΔAIC_LM_ (i.e. difference in AIC between a sigmoidal model and the linear model). VC, visible cycles, PB, perinatal bone present and respective femur length used for growth curve fitting; OC, femur length corresponding to the margin of the outermost cortex was used for growth curve fitting; *N*, total number of femur lengths used for growth curve modelling (VC, VC+1 if perinatal bone or outermost cortex is present, or VC+2 if perinatal bone and outermost cortex are present); MC, number of missing cycles estimated by the growth model. n.a., not applicable; significance levels, *p<0.05, **p<0.01, ***p<0.001.)

taxon	Spec. no.	VC	PB	OC	*N*	model	*L*_0_(mm)	*A* (mm)	*g* (mm yr^−1^)	*t*_*i*_ (year)	
*Psephoderma*	PIMUZ	25	no	no	25	LGM	23.9***	32.9***	0.281***	11.2***	
	A/III 1476					LGM	23.9***	32.4***	0.281***	12.2***	
	PIMUZ	10	no	no	10	LGM	24.8***	57.9***	1.003***	1.4***	
	A/III 0735					vBGM	31.1***	86.7***	0.391***		
						LGM	24.8***	57.9***	1.003***	2.4***	
*Paraplacodus*	PIMUZ T5845 humerus	7	no	no	7	vBGM	48.3***	109.8***	0.391**		
	PIMUZ T5845 femur	10	no	no	10	vBGM	13.8***	101.2***	0.233***		
						vBGM	14.6***	107.0***	0.191***		
P. indet. aff. *Cyamodus*	MHI 1096	6	yes	no	7	LM	36.1**		1.046**		
	MHI 2112–6	4	yes	no	5	LGM	27.4*	206.2*	1.170*	2.5*	
	MHI 697	4	yes	no	5	LGM	20.8*	172.2**	1.317*	1.9*	
	SMNS 15891	6	yes	no	7	LGM	51.4***	125.8***	1.016***	1.9***	
	SMNS 54582	5	yes	no	6	vBGM	32.7**	278.1**	0.184*		
	SMNS 54569	6	yes	no	7	LGM	26.0***	182.0***	0.975**	2.9***	
	SMNS 59831	7	yes	no	8	LM	35.9**		12.929**		
						LGM	22.4***	223.6***	0.661**	3.3**	
	SMNS 15937	7	yes	no	8	vBGM	35.3**	323. 3**	0.140*		
P. indet. group I	IGWH 9	6	yes	yes	8	vBGM	12. 3*	97.0**	0.163*		
	MB.R. 454	5	no	yes	6	LGM	42. 1***	86.3***	0.778**	1.8**	
	IGWH 23	9	no	yes	10	LM	72.0***		0.361***		
						LGM	54.6***	109.1***	0.347***	4.4***	
	SMNS 84545	23	no	no	23	vBGM	68.3***	192.0***	0.110***		
P. indet. group II	MB.R. 814.2	13	yes	no	14	vBGM	75.9**	262.8***	0.214***		
	MB.R. 961	8	yes	no	9	LGM	44.3**	147.5***	1.505***	3.1***	
	MB.R. 812	6	yes	no	7	LGM	24.8***	140.7***	1.256**	1.9***	
*Horaffia kugleri*	SMNS 84816	3	yes	yes	5	vBGM	257.0***	934.6***	0.443*		
	MHI 2112–1	2	yes	yes	4	vBGM	285.8**	844.6**	0.629^+^		
	MHI 2112–2	2	yes	yes	4	vBGM	43.4***	124.1***	1.033*		
	MHI 2112–4	4	yes	yes	6	LM	21.1**		1.103*		

taxon	Spec. no.	MC	Res. s.e.	d.f.	ΔRes_LM_	AIC	d.f.	ΔAIC_LM_	AD (year)	AA (year)	ASM (year)
*Psephoderma*	PIMUZ	0	0.243	21	−0.499	5.9	5	−54.1	25	32–33	11–12
	A/III 1476	1	0.243	21	−0.499	5.9	5	−54.1	26	33–34	12–13
	PIMUZ	0	0.227	6	−0.434	3.2	4	−20.8	10	10–11	1–2
	A/III 0735	0	0.246	7	−0.415	4.7	4	−19.1	10	12–13	0–1
		1	0.227	6	−0.434	3.2	4	−20.8	11	11–12	2–3
*Paraplacodus*	PIMUZ T5845 humerus	0	0.334	4	−0.375	8.2	3	−10.5	7	11–12	0–1
	PIMUZ T5845 femur	0	0.518	7	−0.461	19.0	3	12.7	10	18–19	0–1
		1	0.520	7	−0.460	19.1	3	−12.6	11	19–20	1–2
P. indet. aff. *Cyamodus*	MHI 1096		0.465	5	0	12.8	3	0	6	n.a.	n.a.
	MHI 2112–6		0.505	1	-0.098	10.8	4	-2.1	4	6–7	2–3
	MHI 697		0.032	1	-0.545	-18.4	5	-30.5	4	6–7	1–2
	SMNS 15891		0.308	3	-0.473	7.4	4	-12.6	6	6–7	1–2
	SMNS 54582		0.226	3	-0.332	3.0	4	-10.6	5	16–17	1–2
	SMNS 54569		0.444	3	-0.194	12.6	4	-4.6	6	6–7	2–3
	SMNS 59831		4.651	6	0	51.0	3	0	7	n.a.	n.a.
			4.038	4	-0.613	49.3	4	-1.7	7	10–11	3–4
	SMNS 15937		2.191	5	-1.695	39.5	4	-8.6	7	14–15	1–2
P. indet. group I	IGWH 9		0.277	5	-0.108	6.4	4	-4.2	6	14–15	1–2
	MB.R. 454	0	0.092	2	-0.057	-7.7	4	-5.5	5–6	8–9	1–2
	IGWH 23	0	0.163	8	0	-4.2	3	0	9–10	n.a.	n.a.
		0	0.152	6	-0.011	-4.9	4	-0.7	9–10	19–20	4–5
	SMNS 84545	0	0.135	20	-0.870	-22.2	4	-83.6	23	30–31	0–1
P. indet. group II	MB.R. 814.2		0.155	11	-0.389	-7.8	4	-34.3	13	14–15	0–1
	MB.R. 961		0.199	5	-0.577	1.2	5	-26.0	8	6–7	3–4
	MB.R. 812		0.269	3	-0.436	5.5	4	-13.1	6	6–7	1–2
*Horaffia kugleri*	SMNS 84816		0.243	2	-0.249	3.5	3	-7.1	3–4	10–11	0–1
	MHI 2112–1		0.281	1	-0.255	4.4	3	-5.2	2–3	7–8	0–1
	MHI 2112–2		0.104	1	0.750	-3.5	3	-16.8	2–3	5–6	0–1
	MHI 2112–4		0.137	4	0	-3.2	3	0	4–5	n.a.	n.a.

In the histological record, ages at death can be obscured by remodelling of the inner cortex or by incompleteness of the outermost cortex. Onset of sexual maturity and/or asymptotic length can be obscured in the growth record by the lack of a reduction in growth rate owing to a general irregular cycle thickness or the lack of a distinct change in bone tissue. It is assumed that mathematical growth models indicate sexual maturity by the inflection point (age at which 30% (vBGM) or 50% (LGM) of A is reached) and asymptotic length is always reached at 99%.

## 3. Results

### 3.1 Bone histology of Placodontia

Histologically, the placodont sample can be divided into two groups that do not follow the classical phylogenetic distinction into armoured versus non-armoured Placodontia [1,11–13]. Both the non-armoured *Paraplacodus* and armoured *Psephoderma* grew with lamellar-zonal bone tissue, indicating slow growth rates and a low basal metabolic rate [11,74]. Humeri assigned to Placodontia indet. aff. *Cyamodus* grew with radiating to plexiform fibrolamellar bone [11]. A similar radiating fibrolamellar bone tissue was already described for a single placodont bone by Buffrénil & Mazin [5]. Humeri and femora of Placodontia indet. show fibrolamellar bone with vascular canals arranged circumferentially. Both tissues indicate fast growth rates and probably an increased basal metabolic rate [5,11]. The Placodontia indet. sample can be divided into two histological groups which do not necessarily represent a taxonomical unit [11].

Humeri of Placodontia indet. aff. *Cyamodus*, humerus IGWH 9, as well as femora of Placodontia indet. group II all share an innermost ring of a bone tissue that is quite distinct from the rest of the cortex. It consists of woven bone or poorly organized coarse parallel-fibred bone (figures 3, 4*a*,*e*–*g*, 5 and 6). Mainly simple longitudinal or radial vascular canals and primary osteons are irregularly scattered throughout this tissue. The perinatal bone tissue is separated from the rest of the cortex by a thin layer of highly vascularized bone tissue or a distinct change in bone tissue organization (figures 3, 4*a*,*e*–*g*, 5 and 6). The term ‘perinatal’ is used following Horner *et al*. [75], because it is not known if placodonts gave birth to live young as seen in some other Sauropterygia [76–79] or if their offspring hatched from laid eggs.

### 3.2 Growth records of Placodontia

#### 3.2.1 Psephoderma

**Figure 7.**
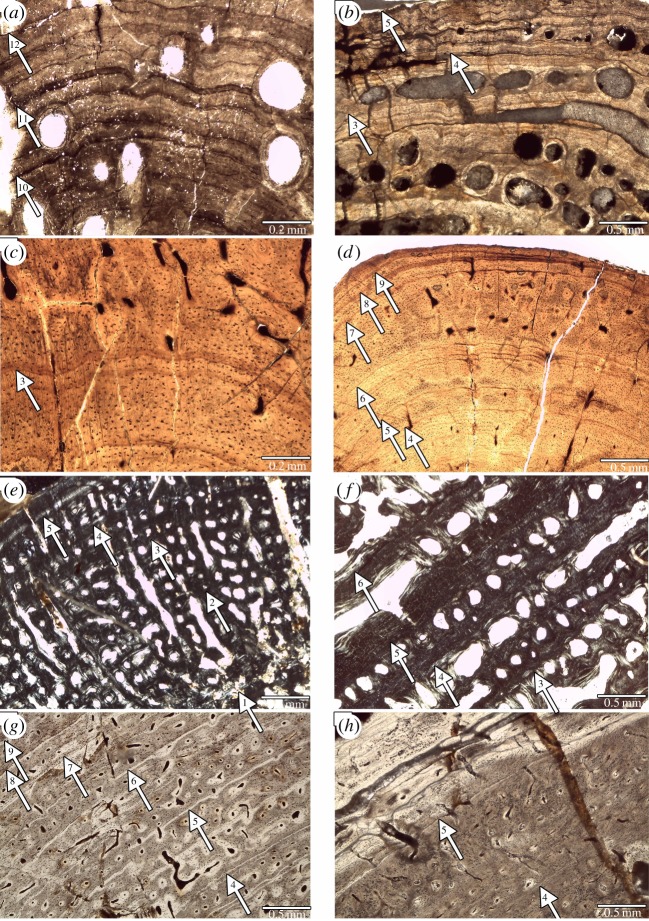
Details of growth marks in Placodontia. Images (*a*–*d*,*g*,*h*) viewed in normal transmitted and (*e*,*f*) in cross-polarized light. (*a*–*d*) Growth marks in lamellar-zonal bone tissue. (*a*) Humerus of *Psephoderma* (PIMUZ A/III 1476). Note the high number of LAGs including parallel, closely spaced double LAGs. (*b*) Femur of *Psephoderma* (PIMUZ A/III 0735). Note the EFS in the outer cortex. (*c*) Humerus of *Paraplacodus* (PIMUZ T5845) showing two pairs of double LAGs. (*d*) Femur of *Paraplacodus* (PIMUZ T5845) showing the triple LAG in the middle of the picture. Note the high number of osteocytes spread throughout the cortex in both *Paraplacodus* samples. (*e*–*h*) Growth marks in fibrolamellar bone tissue in Placodontia indet. (*e*) Humerus MB.R. 454. Arrows mark annual growth marks. (*f*) Femur IGWH 23. Note the lined up arrangement of vascular canals. (*g*) Femur MB.R. 814.2. Note the regular stratification of the cortex by LAGs. (*h*) Femur MB.R. 961. Note the bone tissue change in the outer cortex (arrow with the no. 5).

Histological details are obscured in both *Psephoderma* samples owing to diagenesis and cracks running parallel to the growth marks (figure 7*a*,*b*). The entire primary cortex of the humerus (PIMUZ A/III 1476) is stratified by rest lines (figures 2*a* and 7*a*). Zones and annuli are not distinguishable. The total number of annual growth marks is 25 (table 1), many of which appear in the form of double lines of arrested growth (LAGs) (figure 7*a*). Owing to the large size of the medullary cavity and perimedullary region, at least three growth marks are lost (table 1). The first five visible LAGs are closely and regularly spaced, the following ones are wider but irregularly spaced. From LAG 19 onwards, the LAGs are regularly and closely spaced, resembling an external fundamental system (EFS, *sensu* [80]) or outer circumferential layer (*sensu* [81]). The humerus has two major changes in growth rate preserved (i.e. changes in cycle thickness): after LAG 10 and after LAG 19. In our interpretation, the onset of sexual maturity is indicated after visible LAG 10. Onset of sexual maturity after visible LAG 19 is unlikely because this would be rather late in ontogeny, which is unrealistic in terms of taxon survivorship [82]. The individual had attained its maximum size (i.e. asymptotic length) around visible LAG 19 and continued living for several years until an estimated age of 28 years. Based on its size, the *Psephoderma* humerus represents a half-grown individual [60,83], but based on the number of preserved LAGs and the presence of an EFS it was a fully grown, adult individual.

Up to its mid-part, the cortex of the *Psephoderma* femur (PIMUZ A/III 0735) is divided by equally broad zones and annuli (figures 2*b* and 7*b*). Owing to the size of the medullary cavity, approximately two annual growth cycles are missing. The first and second visible annulus each contains several non-annual rest lines (figure 7*b*). The outer cortex is formed by an EFS containing at least six closely spaced double LAGs (figure 7*b*). Sexual maturity could either have started after visible LAG 3, indicating a decrease in cycle distance, or after visible LAG 5 when no more zones are deposited. The femur indicates that the individual was at least in its 12th year of life when it died.

**Figure 8.**
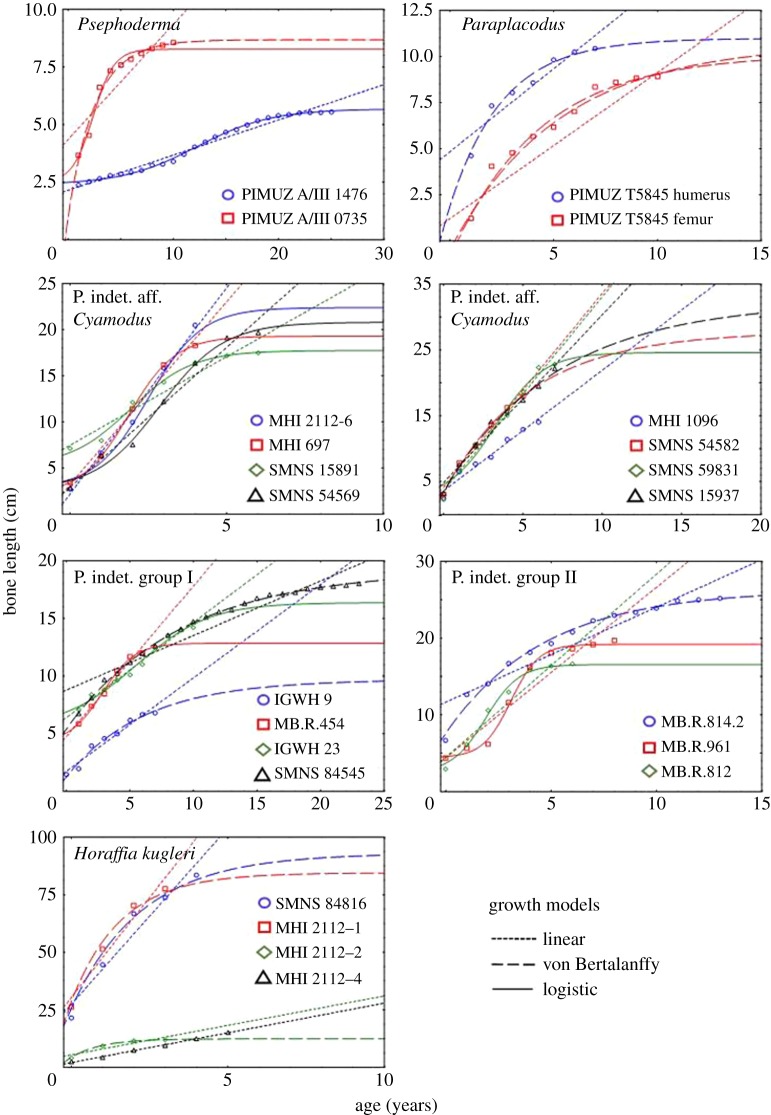
Growth models established for Placodontia. The statistical best growth models are shown for each specimen. The linear model is always plotted to assess whether it is less supported than the sigmoidal model for the specimen (quasi-linear phase of growth, [71]). Parameter values of models and model fitting statistics (*absolute* and *relative* goodness-of-fit of models) are summarized in table 2.

Growth in *Psephoderma* was best described by the logistic growth model (table 2; figure 8). Two equally supported models in terms of AIC were found for the humerus (PIMUZ A/III 1476) that only slightly differ, either assuming that no or only one annual growth mark was missing in the inner bone tissue (table 2). The age at death predicted by these two models are 25 and 26 years, respectively, and the age at which asymptotic length is observed is between 32 and 33 years, and 33 and 34 years, respectively. The inflection point of the two models lies between 11 and 13 years, which agrees with the histological data. Age at death is also in accordance with histology and varies only by about 2 or 3 years. Histological data and growth models for the humerus differ by about 10 years in the age when asymptotic length would have been achieved (tables 1 and 2).

For the femur (PIMUZ A/III 0735), the logistic growth model assumes that no annual growth mark is missing. The age at which the asymptotic length is reached is estimated between 10 and 11 years, which is similar to the histological record (tables 1 and 2). The model reveals an age at sexual maturity between 0 and 3 years and an age at death of around 10 years, whereas histology indicates for both higher ages (table 1).

The much larger *Psephoderma* femur (PIMUZ A/III 0735) has fewer annual growth marks preserved than the humerus, but nevertheless documents a considerable decrease in growth rate. Both the model and the growth record suggest different ages for both bones when sexual maturity starts (tables 1 and 2). Also obvious is the discrepancy between both bones when asymptotic length is achieved, which is also evident from both the histological record and the models. An explanation for this discrepancy between humerus and femur might be high developmental plasticity, or sexual dimorphism in growth patterns in *Psephoderma* [83], which is well documented for other Sauropterygia [4,77,84–86]. Alternative explanations could be the misidentification of bones or erroneous taxonomical assignment.

#### 3.2.2 Placodontia indet. aff. *Cyamodus*

**Figure 9.**
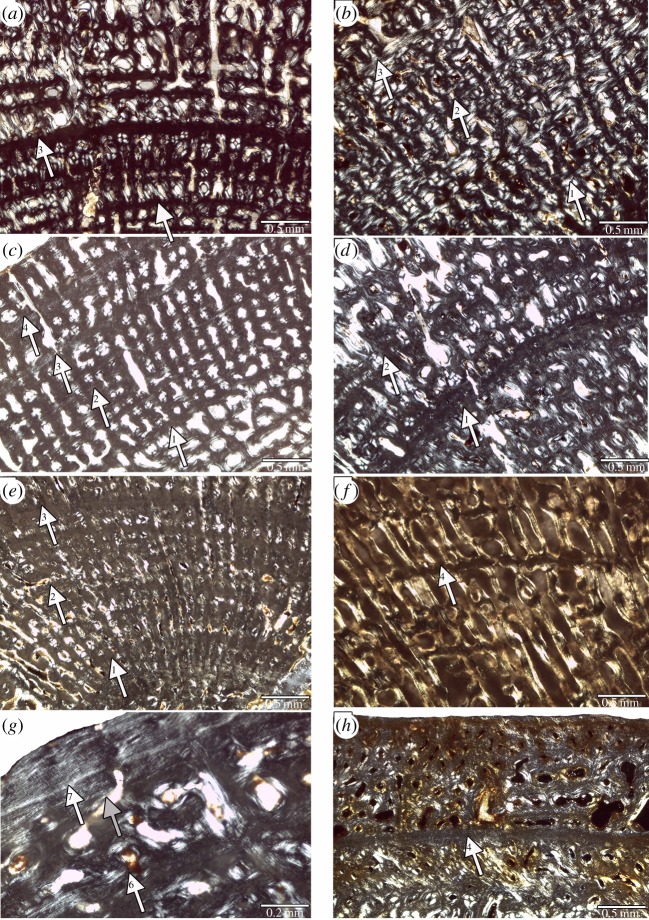
Details of growth marks in fibrolamellar bone tissue in humeri of Placodontia indet. aff. *Cyamodus* (*a*–*g*) and *Horaffia kugleri* (*h*). All images are viewed in cross-polarized light. (*a*) MHI 1096, (*b*) MHI 697, (*c*) SMNS 15891, (*d*) SMNS 54569, (*e*) MHI 2112–6, (*f*) SMNS 15937 and (*g*) SMNS 59831. The grey arrow in (*g*) marks the bone tissue change in the outer cortex. (*h*) MHI 2112–4.

In spite of their fast growth rates and owing to an inhibition of bone remodelling leading to small/reduced medullary cavities, humeri of Placodontia indet. aff. *Cyamodus* have a fairly complete growth record preserved. Growth marks consist of zones, annuli and LAGs in varying combinations. Zones are always broad, whereas annuli are usually very thin (figures 3 and 9). Occasionally an annulus is accompanied by a LAG. In most cases, radial vascular canals dominate the beginning of a zone, whereas at the end of a zone, vascular canal organization changes to a longitudinal organization. Additional stratification of zones results from lined up vascular canals and the presence of subcycles (figures 3 and 9). The postaxial bone side has the thickest cortex and the highest number of subcycles occurs here. Distance between growth cycles strongly varies along the bone sides, depending on cortex thickness.

In most samples, the perinatal bone tissue (figures 3 and 6) is followed by a very broad zone that extends up to the middle cortex. Besides the first cycle, the third cycle is the broadest growth cycle in all samples. Overall growth rate is continuously high in all samples and does not significantly decrease over time. Thus, in none of the samples is a distinct cessation of growth seen (e.g. presence of an EFS). The two largest bones (SMNS 15937, SMNS 59831), probably representing fully grown individuals, show a decrease in cycle distance at the two outer growth cycles and an increase in tissue organization (figure 9*g*). The onset of sexual maturity is neither addressable from changes in cycle thickness nor from bone tissue organization. As most samples show a broad third cycle, one might speculate that onset of sexual maturity was reached during or after this growth cycle.

Growth curve modelling revealed sigmoidal curves for MHI 2112–6, MHI 697, SMNS 15891, SMNS 54569, SMNS 54582 and SMNS 15937, whereas for MHI 1096 a linear model fitted best, and for SMNS 59831 the linear model and a sigmoidal model obtained an equal support in terms of AIC (table 2; figure 8). Except for MHI 1096 and SMNS 59831, in which modelling revealed no reliable information (table 2), modelling of growth clearly splits the humeri into two groups. One group (MHI 2112–6, MHI 697, SMNS 15891, SMNS 54569) followed the logistic growth model, whereas the other group (SMNS 54582, SMNS 15937) followed the vBGM (table 2; figure 8). Both groups achieved onset of sexual maturity between 1 and 3 years. For all samples the growth record consistently indicates ages at death that are 1 year lower than those predicted by the growth models (tables 1 and 2). However, asymptotic length is achieved in the group for which the LGM was best at ages between 6 and 7 years, whereas it is between an age of 14 and 17 years in the two humeri following the vBGM and thus varies strongly (i.e. 10 years). The difference between the asymptotic ages between both groups may indicate a sexual dimorphism in growth pattern or the presence of different taxa within the humeri assigned to Placodont indet. aff. *Cyamodus*.

#### 3.2.3 Paraplacodus

Growth marks in the humerus (PIMUZ T 5845) are deposited in the inner cortex as zones and annuli, whereas LAGs alternate with zones in the middle and with annuli in the outer cortex (figures 2*c* and 7*c*). Many LAGs are double LAGs, one (LAG 4) located at the postaxial bone side even represents a triple LAG (figure 2*c*). Owing to the size of the medullary region, one inner growth cycle might be missing (table 1). The medullary region is surrounded by several indistinct layers of poorly vascularized or even avascular lamellar bone that alternate with vascularized tissue (figure 2*c*), making their interpretation in terms of annual growth cycles difficult. Cycle distance differs strongly at different bone sides (figure 2*c*), precluding its interpretation in terms of onset of sexual maturity. Extrapolation and growth mark count indicates that the individual was in its ninth year of life when it died.

Based on the dimensions of the medullary region, one inner growth cycle is missing in the femur (PIMUZ T5845) (figure 2*d*). Tissue is poorly vascularized and stratified by LAGs, whereas zones and annuli cannot be distinguished. Cycle distance is variable. LAG 6 splits postaxially into four rest lines (figure 7*d*). LAG 9 in the outermost cortex is only locally visible owing to the incompleteness of the outer cortex. An onset of sexual maturity is not detectable in the femur. The femur indicates that the individual was at least in its 10th year of life when it died.

Growth in *Paraplacodus* was best described by the vBGM (table 2; figure 8). For the humerus and the femur (both PIMUZ T5845), the best model assumed that no annual growth marks are missing, which is contrary to the large size of their medullary regions. For the humerus, the vBGM predicts an age at death of 7 years and an age at which asymptotic length would have been reached between 11 and 12 years, and that the individual became sexually mature within its first year of life. Histological age at death is higher than in the model (tables 1 and 2). For the femur, the model estimated an age at onset of sexual maturity within the first year of life and an age at death of 10 years. The predicted age at which asymptotic length was reached is between 18 and 19 years. Although both bones most probably belong to the same individual (see below) growth models vary distinctly in their estimated asymptotic ages.

The size ratio of the humerus and femur of *Paraplacodus*, as well as the discovery circumstances, indicates that both bones could well belong to the same individual (T. M. Scheyer 2014, personal observation). The growth record as well as the growth model provides further evidence for this. The growth record reveals that both bones share a split of one LAG into three (LAG 4, humerus) and four (LAG 6, femur) rest lines at the postaxial side. As is evident from figure 8, both curves can be easily laid together when shifted. The discrepancy in LAG number results from a higher activity of endosteal remodelling and thus from the loss of more inner growth cycles in the humerus. This would mean that in *Paraplacodus*, the femur has the more complete preserved growth record, although it is likely that one inner growth cycle is also lost here. This documents that: (i) growth cycles can have a different combination/sequence and appearance of growth marks in long bones of the same individual (i.e. distinct zones in the humerus versus none in the femur); and confirms that (ii) long bones have different resorption rates as was already documented, e.g. in *Agama stoliczhkana* [87] and *Alligator mississippiensis* [50].

#### 3.2.4 Placodontia indet. group I

The cortex of humerus MB.R. 454 is divided by broad zones and diffuse thin annuli (figures 4*b* and 7*e*). The outermost cortex shows an increase in bone tissue organization. In total, five annuli can be counted, but a new growth cycle has started in the outermost cortex. At least two inner growth cycles are missing (table 1), which would mean that the individual died in its eighth year of life. A clear decrease in growth rate indicating onset of sexual maturity is not documented.

The medullary region of humerus IGWH 9 is surrounded by perinatal bone tissue (figure 4*a*). The end of the first visible growth mark is very distinct. The following cortex is divided by broad zones and thin annuli or LAGs, respectively. After the third growth cycle, vascular density decreases significantly. The outermost cortex shows an increase in bone tissue organization and the annulus deposited here contains several non-annual rest lines. Altogether, six annual growth cycles are counted and the individual died within its seventh year of life. The onset of sexual maturity could be represented by the end of the third cycle, when vascular density decreases.

Zones in femur SMNS 84545 and IGWH 23 often contain only a single row of vascular canals (figures 4*c*,*d* and 7*f*). Annuli are diffuse and not well delimited in SMNS 84545 (figure 4*d*). Its cortex is divided by 15 annual growth cycles before the bone tissue changes into an EFS. The EFS again contains up to eight LAGs, which split and merge. One or two inner growth cycles may be destroyed. This would mean that the individual achieved maximum size around its 16th to 17th year of life, and died at an age of around 25 years. The onset of sexual maturity cannot be determined from histology for SMNS 84545.

The cortex of femur IGWH 23 is divided by nine growth cycles consisting of zones and annuli that each end in an LAG that can be accompanied by several non-annual rest lines. The outermost cortex indicates the start of another zone. Owing to the large medullary region and the short distance between visible growth cycles, the number of reconstructed growth cycles is up to nine. Growth rate decreases after the fourth visible growth mark when vascularization decreases, maybe representing the onset of sexual maturity. The individual died between its 11th and 19th year of life.

Contrary to the histological data, the LGM predicts that no inner cycles are missing for humerus MB.R. 454, which is unreliable owing to the large size of the medullary region. The model further predicts that the individual reached asymptotic length between its 8th and 9th year of life and sexual maturity between the first and second year of life. Both traits are not clearly documented in the histological record.

Growth documented in humerus IGWH 9 and in the femur SMNS 84545 followed the vBGM. Contrary to histological data, the model predicted for SMNS 84545 that no inner growth marks are missing, which is again unreliable. Age at death estimated by the model for IGWH 9 is between 5 and 6 years and at 23 years for SMNS 84545. Asymptotic length is reached at an age between 14 and 15 years for IGWH 9 and between 30 and 31 years for SMNS 84545. The estimated age at sexual maturity is between the first and second year of life for IGWH 9, which is in accordance with the histological record. For SMNS 84545, the onset of reproductive maturity is predicted within the first year of life.

For femur IGWH 23, the linear model and a logistic growth model obtained an equal support in terms of AIC (table 2; figure 8) but both models did not reveal reliable life-history information (table 2).

#### 3.2.5 Placodontia indet. group II

Femora MB.R. 814.2, MB.R. 961 and MB.R. 812 all show remains of perinatal bone tissue (figure 4*e*–*g*). Femora cortex is divided by broad zones and thin distinct annuli containing an LAG and in some cases additional rest lines (figures 4*e*–*g* and 7*g*,*h*).

In MB.R. 814.2, a total of 13 annual growth cycles can be identified and the individual died in its 14th year of life (figure 4*g* and 7*g*). At the postaxial bone side, the outermost LAG splits into three rest lines. Elsewhere at the outer cortex, they are well separated indicating that they indeed represent three different growth marks. The outermost cortex consists of another zone, although decrease in growth seems to be initiated owing to an increase in tissue organization. The onset of sexual maturity could have happened after the third growth cycle owing to a decrease in growth cycle distance or after the seventh growth cycle because zones are no longer deposited.

Femur MB.R. 961 shows five growth cycles before bone tissue organization increases to form an EFS in the outer cortex that again contains at least two LAGs (figures 4*f* and 7*h*). Thus, this individual had achieved maximum size and died shortly after its eighth year of life. After the fourth cycle, cycle distance decreases continuously. In spite of a shorter bone length and the presence of fewer annual growth marks when compared to MB.R. 814.2, MB.R. 961 has an EFS developed in the outer cortex (figure 7*h*). Femur MB.R. 812 shows five annual growth cycles. After the fourth cycle, cycle distance decreases indicating a reduction of growth rate. The outermost cortex consists of a zone and the animal died in its sixth year of life (table 1).

MB.R. 961 and MB.R. 812 followed the LGM, whereas MB.R. 814.2 followed the vBGM (table 2; figure 8). The femora MB.R. 961 and MB.R. 812 achieved asymptotic length at an age between 6 and 7 years. For MB.R. 961, the model predicts the onset of sexual maturity between 3 and 4 years and for MB.R. 812 between 1 and 2 years. For femur MB.R. 814.2, the vBGM reveals that the individual reached its asymptotic length at an age between 14 and 15 years, and sexual maturity is between 1 and 2 years.

Modelling of femora splits them in two groups comparable to that of humeri of Placodontia indet. aff. *Cyamodus*. Modelled ages of femur MB.R. 814.2 fit well to SMNS 54582 and SMNS 15937 and modelled ages of femur MB.R. 961 and MB.R. 812 fit to those humeri of Placodontia indet. aff. *Cyamodus* that followed the LGM. Modelling growth of Placodontia indet. clearly indicates that neither group I nor group II represent a taxonomical union as was suggested before [11] because they are not homogeneous with respect to their growth patterns.

#### 3.2.6 Horaffia kugleri

Humeri of *Horaffia* show an inner ring of perinatal bone tissue (figures 5 and 6*h*). Growth marks consist of broad zones and thin annuli. In each zone, vascularity changes towards its end from a mainly circumferential to a more longitudinal organization that is accompanied by a decrease in vascular density. Each zone contains subcycles. It is noteworthy that the number of growth marks given by Klein & Hagdorn ([69]: table 1) include subcycles and thus do not represent the annual number of growth cycles (table 1).

Humerus SMNS 84816 has a free centre that still contains pockets of calcified cartilage, indicating an early ontogenetic stage (figure 6*h*). The cortex of humerus MHI 2112–1 is highly stratified, and it is very difficult to distinguish annual from non-annual growth marks. MHI 2112–2 has a large medullary region and only few remains of perinatal bone tissue are left. In MHI 2112–4, the third cycle might indicate the onset of sexual maturity. The individual died in its fifth year of life. In none of the bones, a cessation of growth is documented. Except for adult MHI 2112–4, vBGMs best described growth in all *Horaffia kugleri* specimens. The ages at death derived from models match those derived from histology, as perinatal bone tissue is present in these bones (tables 1 and 2). The onset of sexual maturity was within their first year of life according to the models. For SMNS 84816, the growth model predicts an age for reaching asymptotic length between 10 and 11 years, for MHI 2112–1 between 7 and 8 years, and for MHI 2112–2 between 5 and 6 years. For MHI 2112–4, the linear model worked best (although results for this bone are poor). It suggests that the individual died at an age of 6 years during the quasi-linear phase of growth. Both modelling and histological data revealed only limited information on growth for *Horaffia kugleri*, because three of the four bones represent rather early ontogenetic stages (table 1), which is also supported by their bone size.

## 4. Discussion

### 4.1 Overall evaluation

Modelling histological data of Placodontia did not reveal reliable results for all samples. It usually works best for those samples that have a distinct number of annual growth cycles including an early decrease in growth rate (i.e. onset of sexual maturity) and a later decrease in growth rate (i.e. EFS=attainment of asymptotic length) [71]. Thus, in samples of juvenile individuals, modelling is more problematic because they only cover the quasi-linear phase of growth. Nevertheless, except for two samples, modelling worked well for Placodontia indet. aff. *Cyamodus*, which have only a limited number of annual growth cycles and no distinct decrease in growth rate preserved at all. Modelling was not successful for IGWH 23 (Placodontia indet.) and MHI 2112–4 (*Horaffia kugleri*), both of which have a good number of growth cycles preserved.

Congruent growth patterns and growth models may detect taxonomical affinities, as might be the case for humeri of Placodontia indet. aff. *Cyamodus* and femora of Placodontia indet. group II. However, growth models alone are not sufficient to argue that specimens belong to the same taxonomic group because, in extant taxa, growth models are not taxon-specific [57]. Differences in the type of the growth model, specifically in the estimated asymptotic ages between individuals, may point to a sexual dimorphism expressed in different growth traits or may point to the presence of different taxa.

### 4.2 Ageing and maturation in placodonts

The presence of perinatal bone tissue is taxon-dependent, because it was preserved in some placodonts (Placodontia indet. aff. *Cyamodus*, Placodontia indet., humerus IGWH 9, group II; *Horaffia*) but not in all (some Placodontia indet.). The perinatal bone tissue differs from the rest of the cortex owing to a lower organization of tissue and the presence of simple vascular canals. It thus indicates a higher growth rate when compared with the rest of the respective tissues. Changes in growth rate around birth or hatching reflect changes in energy source of the embryo, which is known from modern reptiles and turtles [88]. Although well separated by a change in bone tissue or, in some samples, by a diffuse growth mark, a distinct hatchling line is not present in placodonts and the perinatal bone tissue is always part of the first annual growth cycle. The expression of a hatchling line is highly variable and inconsistent among modern reptiles and does not seem to follow any specific (i.e. environmental or phylogenetic) pattern [36,37,39,89–92].

The very large zone of the first year seen in some placodonts indicates an enormous appositional bone increase, which is consistent with the observation that juvenile extant lepidosaurs can double their size in their first year of life [85,88]. In *Alligator mississippiensis*, apposition rates during the first year of growth are also the highest within its life [50].

The onset of sexual maturity is recorded in the bone tissue of most modern amphibians and reptiles [34,36,54,93]. In most placodonts studied, the onset of sexual maturity is not clearly documented in the bone tissue. This is because of: (i) continuously high growth rates during ontogeny in Placodontia indet. aff. *Cyamodus* and Placodontia indet., (ii) a general irregular cycle distance in all placodonts studied, and/or (iii) owing to the lack of a distinct change in tissue organization in most placodonts studied (table 1). Thus, the onset of sexual maturity was, for most placodonts, deduced from fitted growth models (table 2). Except for the *Psephoderma* humerus (11–13 years, table 2), the onset of sexual maturity was predicted to occur relatively early in ontogeny (0–3 years, table 2), independent of which type of model best fitted to the sample. Such an early onset of sexual maturity is today common among most lepidosaurs, whereas a later onset of sexual maturity is common among crocodiles and turtles [88].

The attainment of maximum size (asymptotic length) is indicated by an EFS in some of the studied placodonts (table 1), independent of bone tissue type. However, in spite of a certain size range (table 1), humeri of Placodontia indet. aff. *Cyamodus* do not show an EFS.

All Placodontia indet. aff. *Cyamodus* humeri show a low total number of annual growth cycles and their resulting age at death in years is lower when compared with, e.g. other placodonts such as *Psephoderma* and *Paraplacodus* or some Placodontia indet. (IGWH 23, SMNS 84545; table 1). For some placodonts studied, modelling revealed an age of over 30 years at which asymptotic length was reached (*Psephoderma*, Placodontia indet. femur SMNS 84545), around 15 years for *Paraplacodus* and some humeri of Placodontia indet. aff. *Cyamodus* and some Placodontia indet., whereas for other placodonts these ages were clearly lower than 10 years (some humeri of Placodontia indet. aff. *Cyamodus* and some Placodontia indet.) (table 2). Thus, some placodonts have a longer maximum lifespan than others or could be related to taxonomy. However, a longer maximum lifespan was not found to be linked to the presence of lamellar-zonal or fibrolamellar bone tissue.

### 4.3 Growth marks

In many modern vertebrates the ‘sharpness’ of growth marks depends on habitat conditions and thus varies between individuals and populations [41]. This is not the case in the Placodontia, where all taxa, independent of locality, stratigraphic age or bone tissue type share strong cyclical growth with well-expressed growth marks. Growth marks include annual growth cycles, numerous non-annual rest lines and subcycles as well as double LAGs, all indicating a strong dependency of growth on exogenous and endogenous factors. Non-annual rest lines and subcycles indicate temporary cessations of growth during the annual cycle, but it is unknown how long they lasted (e.g. days, weeks or months). Irregular cycle distances also indicate strong dependency of growth on exogenous and endogenous factors. Hugi & Sánchez-Villagra [36] reported for the marine iguana *Amblyrhynchus cristatus* that before sexual maturity is reached, zones are thicker than annuli, but after the onset of sexual maturity they are equally broad. However, in placodonts no such pattern is identifiable. Sequences of growth marks are not consistent in placodonts and can change in a single bone as well as among taxa.

Non-annual rest lines have been described for extant animals, such as a newt [70,94] and sea turtles [47]. The presence of splitting growth marks and supplemental lines is also described in *Sphenodon punctatus* [92], *Varanus* spp. [41,42] and *Gallotia* spp. [39]. These lines are always interpreted as a reaction to unfavourable exogenous conditions such as reduced water and/or food availability that often goes along with periods of harsh climatic conditions, forcing the animals to stop or reduce activity. *Triturus marmotus* that lived at a high altitude presumably shows winter and summer dormancy and has double LAGs as well [94]. Circannual activity results in the absence of legible (annual) growth marks [41].

What exactly triggered the high number of non-annual growth marks and double LAGs in placodonts is unclear. Several explanations are conceivable but must remain hypothetical. The presence of double LAGs throughout the placodont sample, which is independent of bone tissue type probably indicates two growth seasons [54] or two reproduction cycles per year. In the Middle Triassic, the Central European Basin was situated within the subtropical convergence zones that had a dry climate with humid intervals [95,96] the latter being influenced by a megamonsoonal climate [97]. Thus, climate was in general warm and water temperatures were high. Dry and rainy seasons produced a distinct seasonality, and several alternating dry and wet seasons may have alternated during a year. This kind of seasonality could well be reflected in the cyclical growth of Placodontia. Alternatively, one could argue that marine vertebrates in a subtropical climate are not particularly influenced by seasonality, because water temperatures seldom fluctuate enough (owing to the high thermic capacity of water) to cause growth cessation. Thus, dropping water temperatures as the main reason for cyclical growth in Placodontia can be largely excluded.

It is also unlikely that lengthy phases of tooth replacement caused dietary stress (and thus induced growth marks), as it has recently been shown that placodonts replaced teeth efficiently and often in unilateral functional groups [15], thus avoiding any prevention of feeding.

The Germanic Basin was always affected by major and minor transgression and regression phases that also influenced salinity, as did storm events and heavy rainfall. It is not known if placodonts possessed salt glands or other osmotic regulators (although this is very likely, since they probably fed underwater), but in any case increased salinity could have caused inactivity and could have affected growth and bone density as well. As a physiological response to periodic changes in salinity, the external periosteal bone of the osteoderms of the temnospondyl *Gerrothorax* was altered by cyclical resorption events [98]. Furthermore, salinity fluctuations [99–101] and storms could have affected food availability by destroying shell banks, one of the major possible food sources of placodonts.

Reproduction activities can also induce cyclicality in growth (e.g. [36]). However, nothing is known about reproduction in placodonts. Viviparity has been documented for several sauropterygian groups (Pachypleurosauria [76,77], a nothosaur [78] and Plesiosauria [79]), but it remains unknown if placodonts were viviparous as well. Lin & Rieppel [85] proposed that males were territorial in the pachypleurosaur *Keichousaurus*, which is also evident for the pachypleurosaurs *Neusticosaurus* spp. and *Serpianosaurus* [8,84,102]. Territorial males often stop feeding for the entire mating period [36], which can last for several weeks and induces growth marks as well. If double LAGs and/or non-annual rest lines are caused by reproduction, they should only be deposited after sexual maturity, which does not apply to all placodonts or is hard to address in our sample, respectively.

### 4.4 Growth patterns

*Psephoderma* and *Paraplacodus* grew with poorly vascularized or even avascular lamellar-zonal bone tissue and thus had low growth rates that are typical also for many modern lepidosaurs [34,54]. Placodontia indet. aff. *Cyamodus* and Placodontia indet. grew with highly vascularized fibrolamellar bone, indicating high, sustained growth rates [11]. Fibrolamellar bone tissue and implied fast growth rates were already described by Buffrénil & Mazin [5] for a placodont humerus. They are also documented for other Sauropterygia such as a pachypleurosaur [6,67], pistosaurs [6,10] and plesiosaurs [10,103], as well as for ichthyosaurs [104,105]. Thus, it has been suggested that extinct marine reptiles had increased growth rates and therefore increased basal metabolic rates when compared with modern ectotherms (including modern marine reptiles) and terrestrial taxa [9,11,105]. A study of oxygen isotope compositions of tooth phosphate of large Mesozoic marine reptiles also ‘suggested high metabolic rates’ [106]. Marine mammals have an increased metabolic rate compared with terrestrial mammals, which is related to osmotic regulation and heat gain [107].

Constant high water temperatures in combination with the high energy/protein of the preferred hard-shelled food of placodonts could have favoured the evolution of fibrolamellar bone tissue in some placodonts. According to Castanet [34], high growth rates are favoured under natural selection in response to external constraints. Thus, one might speculate that placodonts grew so fast to outgrow their predators or became sexually mature very early in their life (as most of them indeed did, see table 2).

It should be emphasized that the *Paraplacodus* and *Psephoderma* samples (lamellar-zonal bone tissue) originated from localities of the Alpine Triassic (lagoonal to shallow shelf environments, with at least partially connected seaways to the Palaeotethys), whereas all the other placodont samples (fibrolamellar bone tissue) come from the Germanic Basin (only shallow marine environments). The latter could have had higher or more constant water temperatures than the first, favouring the development of fibrolamellar bone tissue. In addition, *Paraplacodus* and *Psephoderma* originate from stratigraphically slightly younger localities. However, our sample size is still too small to provide a well-supported conclusion.

Castanet & Baez [39] recorded different growth rates in different species of *Gallotia* spp., where the smallest taxon has the highest and the largest taxon has the lowest growth rate. In Placodontia, the small to medium-sized taxa (*Psephoderma* and *Paraplacodus*) have low growth rates but other small as well as medium-sized and large taxa (Placodontia indet. aff. *Cyamodus*; Placodontia indet. group I and II) show even high growth rates. It is remarkable that bone tissue and vascular density of placodonts growing with radiating fibrolamellar bone [11] are comparable to that of parvipelvian ichthyosaurs, which are sustained swimmers of the open sea [105].

Armoured and non-armoured placodont taxa generally show a high variability in bone tissue [11], as well as in their growth pattern, which both can reflect a strong developmental plasticity. Differences in the type of growth model and differences in estimated asymptotic ages between individuals could thus point to interspecific variation, sexual dimorphism expressed in different growth traits or may point to the presence of different taxa in the sample.

## Data accessibility

All studied bones are stored in a suitable permanent, public repository, which are listed under abbreviations above.
